# Supplementary material for: Analysis of structural and metabolic changes in surface microorganisms following powdery mildew infection in wheat and assessment of their potential function in biological control
Source: PLoS One. 2025 Apr 22;20(4):e0320682. doi: 10.1371/journal.pone.0320682 (PMC12013872; doi:10.1371/journal.pone.0320682)
Supplement: Attachment — Submitted filename: Response to Reviewers.docx [file pone.0320682.s004.docx]

**Response to Reviewers**

(The full text has been polished. The introduction and discussion were rewritten, and biological control was added to the discussion.)

**Reviewer #1:** Xue et al analyzed surface bacterial diversity after wheat infection with powdery mildew and tried to evaluate the potential function of bacteria in biological control. The manuscript is not well-organized and only presents the descriptive results.

（Thank the reviewers for their suggestions. We have reorganized the data of the paper. Please refer to the newly submitted manuscript.）

1. Since powdery mildew can not infect roots of wheat, it is not easy to understand why authors identify the bacteria changes in the roots.

Thank the reviewers for their good suggestions.In the newly submitted paper, all data pertaining to the root research have been removed. The previous analysis, which focused on retaining roots, aimed primarily to observe the response characteristics of both aboveground and underground parts to powdery mildew. However, it was determined that this approach was not effective, and the evidence supporting it was insufficient. Future discussions on this topic are anticipated to emerge from studies of endophytes.

2. Since authors did not evaluate the biocontrol function of the bacteria, it is not suitable mention the information in the title.

The full text and titles have been updated. The part marked in red is the updated content.
3. Authors only showed the differences in the bacterial changes. It is good for readers to know which bacteria are capable of efficiently functioning in biocontrol.

Thank the reviewers for their tips. We have reanalyzed the paper and analyzed and summarized the bacteria for biological control.
4. The introduction and discussion parts should be significantly improved.

Significant improvements have been made to the introduction and discussion.

**Reviewer #2:** Firstly, I appreciate all the authors of this paper for their research plan and work done.

The title of the parer is "Analysis of surface bacterial diversity after wheat infection with powdery mildew and
evaluation of its potential function in biological control." After reading the entire paper I have observed the authors have statistically analyzed surface bacterial diversity in wheat after powdery mildew infection but evaluation of its potential function in biological control is not clear.

Thank the reviewers for their valuable comments. We have analyzed and summarized the metabolic pathways related to biological control and the species with control effect.

The authors have mentioned in Materials and methods under 2.6 Sequence optimization and bacterial function prediction (line no 149), as 'The functional prediction of bacteria refers to these websites: (line no165)
https://www.ncbi.nlm.nih.gov; https://microbewiki.kenyon.edu; https://bacdive.dsmz (line no 165). de/strain (line no 167). And in Results, the details of functional prediction of bacteria are not found. So, clearly show the results of functional prediction of bacteria and explain about it. Shed light on the different evaluation parameters used for functional prediction of bacteria.

Thank you for your comments. The discussion section has been revised, and the results along with the explanations of bacterial function prediction are now presented. Additionally, the various evaluation parameters employed in predicting bacterial function have been clarified.
